# Association between Food Intake, Clinical and Metabolic Markers and DNA Damage in Older Subjects

**DOI:** 10.3390/antiox10050730

**Published:** 2021-05-06

**Authors:** Cristian Del Bo’, Daniela Martini, Stefano Bernardi, Letizia Gigliotti, Mirko Marino, Giorgio Gargari, Tomas Meroño, Nicole Hidalgo-Liberona, Cristina Andres-Lacueva, Paul A. Kroon, Antonio Cherubini, Simone Guglielmetti, Marisa Porrini, Patrizia Riso

**Affiliations:** 1 Department of Food, Environmental and Nutritional Sciences (DeFENS), Università degli Studi di Milano, 20133 Milan, Italy; cristian.delbo@unimi.it (C.D.B.); daniela.martini@unimi.it (D.M.); stefanobernardi@mail.com (S.B.); letizia.gigliotti@unimi.it (L.G.); mirko.marino@unimi.it (M.M.); gargari.g@gmail.com (G.G.); simone.guglielmetti@unimi.it (S.G.); 2 Biomarkers and Nutrimetabolomics Laboratory, Department of Nutrition, Food Sciences and Gastronomy, XIA, INSA, CIBERFES-ISCIII, 08028 Barcelona, Spain; tomasmerono@ub.edu (T.M.); n.hidalgoliberona@ub.edu (N.H.-L.); candres@ub.edu (C.A.-L.); 3 Faculty of Pharmacy and Food Sciences, University of Barcelona, 08028 Barcelona, Spain; 4 Quadram Institute Bioscience, Norwich Research Park, Norwich NR4 7UG, UK; paul.kroon@quadram.ac.uk; 5 Geriatria, Accettazione Geriatrica e Centro di ricerca per l’invecchiamento, IRCCS INRCA, 60127 Ancona, Italy; a.cherubini@inrca.it

**Keywords:** aging, DNA damage, diet, metabolic markers, older subjects

## Abstract

The use of DNA damage as marker of oxidative stress, metabolic dysfunction and age-related diseases is debated. The present study aimed at assessing the level of DNA damage (evaluated as DNA strand-breaks, endogenous and oxidatively-induced DNA damage) in a group of older subjects with intestinal permeability enrolled within the MaPLE (Gut and Blood Microbiomics for Studying the Effect of a Polyphenol-Rich Dietary Pattern on Intestinal Permeability in the Elderly) intervention trial, to evaluate its association with clinical, metabolic and dietary markers. DNA damage in peripheral blood mononuclear cells was assessed by the comet assay in 49 older subjects participating in the study. Clinical and metabolic markers, markers of inflammation, vascular function and intestinal permeability were determined in serum. Food intake was estimated by weighted food diaries. On the whole, a trend towards higher levels of DNA damage was observed in men compared to women (*p* = 0.071). A positive association between DNA damage and clinical/metabolic markers (e.g., uric acid, lipid profile) and an inverse association with dietary markers (e.g., vitamin C, E, B_6_, folates) were found and differed based on sex. By considering the importance of DNA stability during aging, the results obtained on sex differences and the potential role of dietary and metabolic factors on DNA damage underline the need for further investigations in a larger group of older adults to confirm the associations found and to promote preventive strategies.

## 1. Introduction

DNA represents one of the most biologically significant targets of oxidative stress, and it is widely recognised that continuous oxidative damage to DNA may contribute to the development of numerous age-related diseases [1]. Different markers of oxidative DNA damage have been proposed and utilized in numerous studies present in literature [2]. One of the most widely used markers to assess DNA damage is represented by 8-hydroxy-2-deoxy-guanosine (8-OHdG) [3,4]. This marker can derive from oxidative damage and excision-repair, oxidation of free bases or nucleotides, but also from other nucleic acids. Free 8-OHdG can be determined in different biological samples such as blood, urine and tissues; however, urinary 8-OHdG does not directly reflect DNA oxidation within cells [5]. Another measurement of DNA damage can be performed through the use of comet assay [6,7]. The comet assay represents one of the most popular, versatile, simple, sensitive and non-invasive electrophoretic techniques able to detect, in individual cells, different types of damage (e.g., single and double-strand DNA breaks, alkali-labile lesions, DNA–DNA/DNA–protein cross-links) [8]. The comet assay is widely used in numerous in vitro and in vivo studies including observational and dietary intervention studies [9]. Measures of DNA damage using the traditional comet assay, which detect DNA strand breaks by the appearance of tailing, are not specific for oxidative damage. A direct measurement of oxidative damage to DNA (i.e., oxidised DNA bases) can be obtained in vivo by using modifications of the comet assay [10]. The most common modifications include the use of specific enzymes such as endonuclease III for the evaluation of oxidised pyrimidines, and formamidopyrimidine DNA glycosylase (FPG) for the detection and removal of the oxidatively damaged purines (e.g., 8-OHdG). This procedure allows a more direct measurement of DNA damage within the cells and a quantitative comparison with appropriate control [10,11]. A further comet protocol often utilised enables the evaluation of DNA susceptibility to oxidative damage generally induced by using H_2_O_2_. This assay can give further information on cell capacity to counteract an oxidative stress [9].

It has been suggested that there is an association between age and DNA damage [12]. Specifically, several factors can contribute in increasing the levels of DNA damage, from both intrinsic and extrinsic sources [13]. Aging is considered as a progressive and biological phenomenon that leads to loss of physiological integrity and to an impairment of numerous functions, at the molecular, cellular, tissue and organ level [12,13]. Several causes of aging have been hypothesized including an exacerbation of oxidative stress attributed to an imbalance between oxidant molecules and the antioxidant defence mechanisms [13]. This condition brings an accumulation of damage to macromolecules such as DNA but also carbohydrates, lipids, and proteins [14]. In this regard, one important aspect of the ageing process is the progressive accumulation of DNA damage over time [12]. A phenomenon strictly correlated with the aging process is inflammation. A greater presence of pro-inflammatory factors, such as interleukins (e.g., interleukin-6, IL-6; interleukin-1beta, IL-1β) and cytokines (tumour necrosis factor alpha, TNF-α) has been observed in older subjects contributing to generate a persistent and prolonged low-grade inflammation phenomenon called “inflamm-aging” [15]. Additionally, during inflammatory response, reactive oxygen and nitrogen species are produced to combat pathogens and to stimulate tissue repair and regeneration; however, these substances can also damage DNA and induce mutations.

Among the external factors that may contribute to the onset and progression of oxidative stress, and thus on DNA damage, diet and its components can play a pivotal role [16]. Several essential nutrients such as vitamins (e.g., folate, vitamin B12, niacin, vitamin C and E), minerals (e.g., magnesium, zinc, iron, manganese, selenium), and non-essential nutrients such as phytochemicals (e.g., polyphenols, carotenoids, and other bioactives) constitute a fundamental pillar for the preservation of DNA integrity due to their numerous important biological activities [17,18,19,20,21,22,23]. In fact, they are required for numerous functions including nucleotide synthesis, DNA replication, maintenance of DNA methylation, chromosome stability, prevention of DNA oxidation, DNA damage repair [24,25]. On the other hand, excessive energy intake, mainly from fats and in particular from saturated fatty acids, may bring to overweight/obesity and to increased levels of DNA damage also related to an alteration of the repair mechanisms [26]. This is quite common in older subjects in which physiological-social and environmental factors, oral-dental-deglutition disorders, dementia, long drug treatments can compromise the nutritional status thus potentially affecting DNA stability. In particular, several studies have reported that older subjects have higher levels of DNA damage compared to younger individuals [27,28].

To date it is not possible to identify a predominant factor responsible for the levels of DNA damage in specific target groups, but it can be assumed that the damage may derive from a combination of multiple factors that directly or indirectly affect DNA stability. In this regard, it has been suggested that increased intestinal permeability (IP), promoting the translocation of inflammogenic factors could contribute to increase oxidative stress and inflammation, thus representing a further potential factor to control [29].

The main aim of the present study was to assess the levels of DNA damage in a group of older subjects with increased intestinal permeability enrolled in the MaPLE (Gut and Blood Microbiomics for Studying the Effect of a Polyphenol-Rich Dietary Pattern on Intestinal Permeability in the Elderly) study. The association of DNA damage with clinical, metabolic and dietary markers has been also investigated in order to identify the potential relationships in this target group.

## 2. Materials and Methods

### 2.1. Setting and Subjects’ Recruitment

Fifty-one subjects completed the MaPLE trial carried out at Civitas Vitae (OIC Foundation, Padua, Italy), an institution including residential care and independent residences for older subjects [30]. Subjects were selected based on different inclusion and exclusion criteria as previously reported. Briefly, subjects had to be ≥60 years old, with a good cognitive and nutritional status, and a general good health condition while presenting an increased IP evaluated as serum zonulin level [30]. Celiac disease, major renal, liver or respiratory dysfunctions were considered as exclusion criteria together with antibiotic treatment or malignant tumour in the near previous period. Volunteers recruited were non-smokers and information on past smoking habits was not collected. The MaPLE study protocol was approved by the Ethics Committee of the Università degli Studi di Milano (15 February 2016; ref.: 6/16/CE_15.02.16_Verbale_All-7) while the trial was registered in a public register (28 April 2017; ISRCTN10214981; http://www.isrctn.com/ISRCTN10214981, accessed on 15 April 2021). Each participant received detailed information about the study purpose and procedures. Specifically, the MaPLE trial was aimed at demonstrating the role of a polyphenol-rich dietary pattern in the reduction of intestinal permeability and improvement of metabolic phenotypes in older adults. A written consent was obtained by each volunteer after acceptance. The full experimental design and inclusion and exclusion criteria were previously reported [30]. For the present study, the characteristics of subjects at baseline have been considered.

### 2.2. Food Intake

Food intake was collected through the analysis of weighed food diaries. In particular, subjects were asked to provide a food diary the day before the blood drawing. Data obtained were compared with those collected along the whole study in order to verify reproducibility of food habits of the volunteers. This was ensured thanks also to the moderate variety of the meals available within the standard menu provided at the nursing home [31]. The full protocol has been reported in a previous manuscript [30]. Energy, nutrient and polyphenol (single classes and total polyphenols assessed by Folin-Ciocalteu method) intake were estimated through the Metadieta software (Me.Te.Da S.r.l., Rome, Italy) and Phenol-explorer (phenol-explorer.eu).

### 2.3. Anthropometric, Clinical and Metabolic Makers Analysis

Weight, height, body mass index (BMI) and blood pressure were analysed according to international guidelines of Lohman et al. [32] and the Joint National Committee on Prevention, Detection, Evaluation, and Treatment of High Blood Pressure guidelines (JNC 7) [33], as previously described. Blood samples were collected after an overnight fasting for the analysis of a plethora of markers [30]. Plasma and serum were obtained as previously reported [30] and stored at −80 °C until analysis. Metabolic markers (i.e., glycaemia, insulin, lipid profile, liver and renal function) were evaluated in serum through a validated protocol, using an automatic biochemical analyser (ILAB 650, Instrumentation Laboratory, Lexington, MA, USA). Inflammatory (e.g., IL-6, TNF-α, C-reactive protein (CRP) and vascular function markers (i.e., vascular cell adhesion molecule 1, VCAM1; intercellular adhesion molecule 1, ICAM1) were analysed in serum through ELISA kits (R&D System, Minneapolis, MN, USA). Finally, serum zonulin levels, as marker of IP, were quantified using the Immunodiagnostik ELISA kit (Bensheim, Germany) [34].

### 2.4. Isolation of Peripheral Blood Mononuclear Cells

Peripheral blood mononuclear cells (PBMCs) were separated by using Histopaque 1077 density gradient, according to the procedure reported by Del Bo’ et al. [35]. Isolated PBMCs were then diluted into a medium constituted by fetal bovine serum, RPMI 1640 and dimethyl sulfoxide (50:40:10) and stored at −80 °C until analysis. The analysis of DNA damage was performed on PBMCs after a rapid thawing at 37 °C followed by a washing step with fresh RPMI medium and cold phosphate buffer saline. For the analysis, both endogenous and oxidatively-induced DNA damage were evaluated by comet assay [35]. The analysis was performed on samples at baseline.

### 2.5. Analysis of DNA Damage by the Comet Assay

The evaluation of endogenous DNA damage as formamidopyrimidine DNA glycosylase (FPG) sensitive sites was carried out enzymatically by the use of FPG able to detect the oxidized purines (mainly 8-oxo-7,8-hydroxyguanine). A description of the specific steps and conditions of the protocol used has been previously reported [35]. In brief, a solution of low melting point agarose was added to PBMCs suspension, mixed and rapidly spotted onto GelBond Film (VWR International U.R.L., Monroeville, PA, USA) precoated with normal melting point agarose. Coverslips were added on top of the slides and left to solidify for a few minutes at 4 °C. After solidification, coverslips were removed and slides were transferred into a lysis buffer for 1 h at 4 °C in the dark. Then, slides were washed 3 times in a cold buffer and further processed. One slide was treated with a buffer solution containing the FPG enzyme, while the other slide (control) with the same buffer without FPG. Samples were then incubated at 37 °C for 45 min. Successively, the slides were transferred in a horizontal electrophoresis tank containing an alkaline electrophoresis buffer for 40 min at 4 °C in order to favour DNA unwinding, followed by an electrophoresis step (25 V, 300 mA, 20 min). Finally, samples were washed in a neutralizing buffer for 15 min at 4 °C in the dark and dried in ethanol for 2 h.

Oxidatively induced DNA damage was assessed according to the procedure previously reported [35]. Briefly, two GelBonds Film containing cell suspensions were prepared for each subject: one was treated with hydrogen peroxide (H_2_O_2_ 500 µM, 5 min) in a buffer solution at room temperature in the dark; the other was treated for 5 min only with the buffer solution (control slide). Following the oxidative treatment, slides were immersed in a lysis buffer for 1 h at 4 °C in the dark, and then transferred in a horizontal electrophoresis tank and treated as previously reported for the evaluation of endogenous DNA damage.

Ethidium bromide was used for the staining process. At least fifty comets per gel (i.e., 100 comets per condition) were scored using an epifluorescence microscope and analysed with an image analysis system (Cometa 1.5; Immagini e Computer, Bareggio, Milan, Italy). The levels of DNA damage were calculated as tail intensity (% DNA in tail).

### 2.6. Statistical Analysis

Values were reported as mean ± standard deviation (SD), median and interquartile range (IQR, 25–75° percentile). Statistical analyses were performed by means of STATISTICA software (Statsoft Inc., Tulsa, OK, USA). Normality was assessed by the Shapiro–Wilk test. Significant differences at baseline based on sex were determined by unpaired Student’s t test. The regression and correlation analyses (Spearman and Kendall test) were carried out to highlight associations between the levels of DNA damage and dietary, clinical and metabolic parameters in the whole group of subjects and in women and men. In addition, volunteers were stratified according to clinical variables in quartiles, and significant differences in DNA damage biomarkers in each quartile were assessed by unpaired Student’s *t* test. Significance was set at *p* ≤ 0.05; significance in the range 0.05 < *p* < 0.10 was considered as a trend.

## 3. Results

### 3.1. Subject Characteristics

Supplementary Table S1 reports the characteristics of 49 subjects (22 men, 27 women) enrolled within the MaPLE intervention study. The full data set has been already reported [34]. Briefly, subjects analysed ranged between 60 and 98 years old with a median value of 77 years. A high inter-individual variability was shown for BMI (IQR: 22.7–30.7), glucose levels (IQR: 87–113), total cholesterol (IQR: 167–237) (Table S1). Similar findings were observed for the levels of FPG-sensitive sites (IQR: 21.4–34.9) and H_2_O_2_-induced DNA damage (IQR: 9.0–24.0) also after stratification based on sex (Table 1). On the whole, data on DNA damage were comparable between groups. Only a trend towards higher, but not significant (*p* = 0.071), levels of FPG-sensitive sites was documented in men compared to women.

### 3.2. Correlation between DNA Damage and Dietary Markers

Supplementary Table S2 reports the data obtained for dietary markers in the 49 out of 51 subjects enrolled within the MaPLE intervention study. The full data set has been already reported [34]. In Table 2 are shown the correlations between the levels of DNA damage and the dietary markers (energy, macro and micronutrients, and polyphenols).

Regarding the levels of FPG-sensitive sites, an overall negative correlation was found in the whole group of subjects for the intake of vitamin C (*p* = 0.03), vitamin E (*p* = 0.008), vitamin B_6_ (*p* = 0.002) and folates (*p* = 0.008), while no significant correlations were evidenced for the rest of macro and micronutrients analysed. In addition, no association was also found when considering polyphenols and their subclasses.

As regards the levels of H_2_O_2_-induced DNA damage, an overall positive correlation was reported in the whole group of subjects with the intake of cholesterol (*p* = 0.003), omega 3 fatty acids (*p* = 0.021) and vitamin B_6_ (*p* = 0.032), while no association emerged for the other dietary markers including polyphenols and the different subclasses analysed.

The analysis performed stratifying subjects based on sex (Table 3) did not show any significant correlation between the levels of DNA damage (both FPG-sensitive sites and H_2_O_2_-induced DNA damage) and the majority of the dietary markers. Regarding the levels of FPG-sensitive sites, a negative correlation was found with the intake of folates and vitamin B_6_ (*p* = 0.004; *p* = 0.046, respectively) in women, and the intake of vitamin E (*p* = 0.042) in men. In addition, a negative correlation with the intake of monounsaturated fatty acids (MUFA; *p* = 0.048) was observed. No association was observed for polyphenols and their subclasses, apart from an apparent, but not significant (*p* = 0.052) inverse correlation found in men. Regarding the levels of H_2_O_2_-induced DNA damage, a positive correlation with dietary cholesterol (*p* = 0.010) and a negative correlation with total fibre intake (*p* = 0.049) was found in women, while in men no correlation was evidenced with the dietary markers considered including with the intake of polyphenols and their subclasses.

### 3.3. Correlation between the Levels of DNA Damage and Anthropometric, Metabolic and Clinical Markers

In Table 4 the correlations between the levels of DNA damage and anthropometric, metabolic and clinical markers are reported. Despite not being significant (*p* = 0.086), an inverse association between the levels of FPG-sensitive sites and age was observed. On the whole, no correlation was found between FPG-sensitive sites and metabolic and clinical markers neither in the whole group of subjects nor following sex stratification (Table 5).

As regards the levels of H_2_O_2_-induced DNA damage (Table 4), a negative correlation was found with weight (*p* = 0.034), BMI (*p* = 0.042), diastolic blood pressure (DBP: *p* = 0.022), aspartate transaminase (AST: *p* = 0.031) and alanine transaminase (ALT: *p* = 0.011). Interestingly, the distribution of data in quartiles showed a lower level of DNA damage in subjects with the highest BMI, compared to those with the lowest BMI. Conversely, the subjects with the highest low density lipoprotein/high density lipoprotein ratio (LDL/HDL ratio) showed the highest DNA damage (Supplementary Figure S1). Stratification on the basis of HOMA index and CRP showed only a trend towards an increase of H_2_O_2_-induced DNA damage between the first and fourth quartile. No correlation was found for any of the other markers analysed.

After sex stratification (Table 5), a positive correlation between the levels of H_2_O_2_-induced DNA damage and both uric acid (*p* = 0.042) and LDL/HDL ratio (*p* = 0.035) were found for women, while a negative correlation was observed with BMI (*p* = 0.032). Regarding men, statistical analysis revealed a negative correlation between the levels of H_2_O_2_-induced DNA damage and DBP (*p* = 0.039) and ALT (*p* = 0.035). No correlation was shown for the rest of the markers under study both in men and women.

## 4. Discussion

The aging process can be considered a significant determinant of DNA damage levels due to increased oxidative stress, inflammation and other age-related conditions. However, available studies do not always report univocal results, in particular when DNA damage is evaluated by using the comet assay [9,36,37,38].

In the present study, we could not demonstrate a significant correlation between age or sex on DNA damage. However, it was observed a trend towards an inverse association between the levels of FPG-sensitive sites (endogenous DNA damage) and age, and a higher (but still not significant) level of damage in men compared to women, we may attribute to the limited sample size analysed.

As regards the discrepancy among the results found in literature on the topic, it could be related to many potential confounding factors. In this regard, the findings of a recent review showed that lifestyle factors, including diet, and other external exposures may contribute to the damage during aging more than age and sex itself [39].

Diet and dietary components have been largely studied for their modulatory effects on numerous biological functions including the protection against oxidative stress. These factors can contribute to the positive/negative modulation of DNA damage during the aging process. In our study, one of the main results observed regards the inverse association between dietary fibre and vitamins and the levels of DNA damage, and the positive association found with dietary lipids and cholesterol. A plethora of research has reported a positive and/or inverse association between DNA damage and specific dietary patterns [40,41] and/or different macro/micronutrients intake [9,16]. For example, the amount of dietary fats and calories has been reported to play a role in the modulation of oxidative DNA damage levels. In fact, high dietary fat consumption contributes to free radical-induced lipid peroxidation causing damage to macromolecules, including enzymes and DNA [16]. In particular, the intake of saturated fatty acids (SFAs) appeared to be an important determinant of basal DNA damage, on the contrary monounsaturated fatty acids (MUFAs) and polyunsaturated fatty acids (PUFAs), specifically omega-3, have been associated with a reduced level of damage [16,42].

In our experimental conditions, the subjects enrolled had an overall good nutritional status [34]; in addition, there were no significant differences between women and men for any of the dietary variables evaluated [31]. This is in part due to the homogenous food choices related to the availability of meals provided through the daily menu at the nursing home. However, we found a positive association between the intake of dietary cholesterol and the levels of oxidatively induced-DNA damage. This positive association was also confirmed when considering differences according to sex, in particular a positive association with total cholesterol was observed in women while an inverse association with MUFA in men.

As regards dietary fibre, it plays a key role in human health and its intake is associated with a reduction in weight gain, the incidence of diabetes, obesity prevalence, hypercholesterolemia, inflammation and indirectly also reduced oxidative stress [43,44]. Overall, the intake of fibre in our population was relatively low (i.e., about 17–18 g/day) [31] with respect to dietary recommendation (at least 25 g/day) defined by Italian and international guidelines. Statistical analysis did not show any inverse association between total fibre intake (adjusted for energy intake) and the levels of DNA damage. However, when considering data stratified by sex, an inverse association was found in women.

Also vitamins and minerals can contribute in the modulation of activities against DNA damage [25]. For example, the intake of vitamin C and vitamin E has been found inversely associated with the levels of 8-oxodG and DNA strand breaks [45]. Similarly, folates, vitamin B_6_ and B_12_ have been associated with lower risk of single and double DNA strand breaks [45,46,47,48]. Among minerals, magnesium, calcium, iron, copper, zinc and selenium have been found inversely associated with DNA damage [49,50] probably due to their role in the functioning of numerous antioxidant enzymes [25]. However, other studies have found a positive correlation between high calcium and iron intake and DNA damage [51,52]. In our trial, we did not underline critical vitamin and mineral intakes [31]. However, interestingly, we have found an inverse association between the levels of FPG-sensitive sites and the intake of vitamin C, vitamin E, folates and vitamin B_6_, while no correlations were found with the intake of minerals. The inverse associations were also confirmed when stratifying subjects by sex further underlying the importance of adequate vitamin intake in this target population.

Besides macro and micronutrients, also the protective effect of bioactives such as polyphenols against oxidative stress has been largely investigated [18]. A number of studies have shown the capacity of these compounds to neutralize the harmful effect of reactive oxygen species as well as to disrupt their propagation [53,54]. Furthermore, several studies have reported their ability to increase the activity of numerous antioxidant enzymes (e.g., catalase, superoxide dismutase, glutathione peroxidase) and to upregulate nuclear factor erythroid-related factor and antioxidant-responsive elements, crucial for the modulation of oxidative stress and thus DNA damage [53,54]. In this regard, a recent review has documented the capacity of polyphenols to protect cells from DNA damage suggesting it could be dependent on the type and amount of polyphenols tested, especially in in vitro studies where the doses administered can be very high with respect to an in vivo situation [48]. In fact, several in vitro studies reported an apparent increase in the levels of DNA damage after supraphysiological concentrations of polyphenols.

The contribution of polyphenols in the modulation of DNA damage has been documented also in numerous human trials [55,56] showing a decrease in endogenous and oxidatively induced-DNA damage after administration of dietary doses of polyphenols and/or polyphenol-rich foods in different target group of population [57,58,59,60].

In the present study, we estimated a total dietary polyphenol intake of about 660 mg/day (data based on Folin–Ciocalteau analysis as reported in the Phenol-Explorer database and other literature) as previously reported [31], with flavonoids and phenolic acids as the most consumed polyphenol classes. The intake was comparable between women and men and in line with other studies performed in the Italian population (In Chianti study) [61], but apparently lower if compared to the intake of other target populations [57]. Moreover, we could not find any significant association between total polyphenols or subclasses intake, and the levels of DNA damage, apart from an apparent, but not significant, inverse correlation in men. It is also noteworthy that we could not demonstrate in our group of older subjects the protective effect of these bioactive compounds against DNA damage even following a polyphenol rich dietary pattern [34].

In our study, we also tried to identify the possible relationship between anthropometric, clinical and metabolic markers, and the levels of DNA damage. It is widely recognized that metabolic and clinical markers such as glycaemia, insulin, lipid profile, blood pressure together with inflammatory markers (e.g., CRP, IL-6, TNF-α), as well as body weight and BMI, may represent important determinants in the onset and/or progression of oxidative stress [9]. As previously reported, these variables were in the normal range in our target group apart from an overall mild overweight (mean BMI 27 kg/m^2^) [34].

We did not evidence associations between endogenous DNA damage, as FPG-sensitive sites, and the markers under evaluation, even if an apparent trend for age (*p* = 0.086) was underlined. On the contrary we have found an unexplained inverse association between the levels of H_2_O_2_-induced DNA damage (as marker of cell resistance to oxidative stress) and BMI, body weight and DBP while only a trend towards a positive association with SBP, LDL/HDL ratio and CRP. After sex stratification, the associations seemed to be sex-specific (e.g., the inverse correlation with renal/liver function enzymes, DBP, body weight and BMI). Despite some cross-sectional studies that have reported results in line with our findings, a large number of human trials documented a positive association between overweight/obesity and DNA strand breaks or oxidative DNA damage [45,62,63]. A possible explanation for these conflicting results could be related to the cryopreservation process that could have activated a further metabolic activity in the frozen cells that make them more resistant when exposed to an oxidative stress condition, as already reported [35]. In addition, we may hypothesize that the inverse association found between the levels of H_2_O_2_-induced DNA damage and body weight and BMI could be related to catalase activity. This enzyme is involved in the conversion of H_2_O_2_ to hydrogen and water. Some studies have reported higher catalase activity in overweight/obese subjects compared to lean individuals as a compensatory mechanism to counterbalance an oxidative stress condition and an increase in the metabolic production of H_2_O_2_ in these subjects [64,65]. Although this could partially explain the inverse correlation found between DNA damage and BMI, the lack of data on enzymatic activity and the mild overweight of the subjects makes this consideration only a speculation.

Regarding the other metabolic markers under study, a positive association was depicted with LDL/HDL ratio (significant) and total cholesterol/HDL ratio (not significant) in women, supporting the contribution of dietary and circulating lipids in the increase of DNA damage as also reported in literature in different target groups [42,66,67]. Another interesting positive correlation was observed between the levels of uric acid and DNA damage evaluated following the ex vivo induced oxidative stress. Uric acid is the major endogenous end-product of purine metabolism. Experimental and clinical evidence suggests that uric acid could play an important role as antioxidant and could be involved in DNA repair mechanisms [68,69]. However, at the same time, high levels of uric acid were recognized as a marker of acute, severe and chronic inflammatory states [70,71]. In addition, evidence exists, mainly based on epidemiological studies, that high uric acid levels can be considered as an important risk factor for oxidative stress and may contribute to an early onset of cardiovascular, renal and metabolic diseases [72]. It is worth noting that in our population the levels of this marker were within the normal range, thus these findings should be further investigated to support the significance of the association in the older subjects also in view of recent published results [73].

Finally, in the present investigation we could not evidence any relationship between serum zonulin levels, evaluated as a marker of IP, and the different DNA damage markers analysed. The selection of subjects based on their increased IP and small sample size could justify the lack of significant relationships. This result cannot be considered conclusive since no other data have been previously published, although it may be considered plausible that an increased IP, often linked to age related dysbiosis, could promote higher oxidative stress [74,75].

The results obtained in the present study are preliminary and the overall approach has some strengths related to the type of population under study and the specific setting enabling the control of numerous variables, including diet. However, some limitations are worth to be highlighted. First of all, the number of subjects enrolled does not allow a definitive conclusion above all by considering the high inter-individual variability in DNA damage levels found in our study and reported also in the literature. In addition, the lack of an actual analysis of nutritional status through adequate biomarker of intake could represent a further limitation.

## 5. Conclusions

In conclusion, the results obtained support the association among some dietary and clinical/metabolic markers and the levels of DNA damage in older subjects. However, further investigations are needed to confirm the association found in a larger group of older individuals in which also the impact of other confounding factors should be considered.

## Figures and Tables

**Table 1:** Levels of DNA damage markers evaluated at baseline.

Marker of DNA Damage	All (*n* = 49)	Men (*n* = 22)	Women (*n* = 27)	*p*-Value
FPG-sensitive sites (% DNA in tail)	16.5 ± 9.0	18.6 ± 10.4	14.8 ± 6.8	0.071
H_2_O_2_-induced DNA damage (% DNA in tail)	28.7 ± 11.4	28.5 ± 11.6	29.0 ± 2.4	0.438

Data are reported as mean ± SD (standard deviation); *p* < 0.05 are significantly different between groups. FPG, formamidopyrimidine DNA glycosylase.

**Table 2:** Correlation analysis between dietary markers and DNA damage in the whole group of subjects.

Dietary Markers	FPG-Sensitive Sites			H_2_O_2_-Induced Damage		
	Tau	*Z*	*p*-Level	Tau	*Z*	*p*-Level
Energy (kcal)	0.064	0.650	0.516	0.023	0.234	0.815
Total Carbohydrates (% of energy)	0.021	0.217	0.828	−0.032	−0.322	0.748
Simple Carbohydrates (% of energy)	−0.026	−0.261	0.794	−0.019	−0.191	0.849
Proteins (% of energy)	−0.016	−0.167	0.867	−0.032	−0.325	0.745
Animal Proteins (% of energy)	−0.067	−0.675	0.500	0.051	0.517	0.605
Vegetal Proteins (% of energy)	0.013	0.135	0.893	0.038	0.386	0.700
Total Lipids (% of energy)	−0.048	−0.488	0.626	0.082	0.827	0.408
SFA (% of energy)	−0.069	−0.699	0.485	0.113	1.143	0.253
MUFA (% of energy)	−0.137	−1.387	0.165	0.099	1.000	0.317
PUFA (%of energy)	−0.056	−0.571	0.568	0.061	0.616	0.538
ω-6 (%of energy)	−0.112	−1.131	0.258	0.082	0.835	0.404
ω-3 (%of energy)	−0.101	−1.027	0.305	0.114	1.159	0.247
Total Fibre (g/1000 kcal)	−0.084	−0.849	0.396	−0.108	−1.093	0.274
Cholesterol (mg)	0.006	0.061	0.951	0.289	2.929	**0.003**
Calcium (mg)	−0.022	−0.225	0.822	0.038	0.381	0.703
Iron (mg)	0.157	1.596	0.110	0.016	0.158	0.875
Vitamin B_12_ (mcg)	−0.002	−0.019	0.985	0.161	1.632	0.103
Vitamin C (mg)	−0.294	−2.978	**0.003**	0.123	1.245	0.213
Vitamin E (mg)	−0.262	−2.653	**0.008**	0.083	0.843	0.399
Vitamin B_1_ (mg)	−0.100	−1.012	0.312	0.008	0.076	0.939
Folates (mcg)	−0.310	−3.138	**0.002**	0.153	1.546	0.122
Vitamin B_6_ (mg)	−0.263	−2.670	**0.008**	0.212	2.147	**0.032**
Flavonoids (mg)	0.167	1.692	0.091	0.005	0.052	0.959
Lignans (mg)	−0.031	−0.315	0.753	0.149	1.508	0.132
Other polyphenols (mg)	−0.135	−1.370	0.171	0.124	1.255	0.210
Phenolic acids (mg)	−0.005	−0.052	0.958	0.012	0.122	0.903
Stilbenes (mg)	0.012	0.122	0.903	−0.039	−0.395	0.693
Total Polyphenols (mg)	0.138	1.401	0.161	−0.032	−0.328	0.743
TPC Folin (mg)	−0.024	−0.242	0.809	−0.055	−0.553	0.580

Legend: Regression and correlation analyses obtained by Spearman and Kendall test. Data reported in bold are statistically significant (*p* < 0.05); FPG, formamidopyrimidine DNA glycosylase; SFA, saturated fatty acids; MUFA, monounsaturated fatty acids; PUFA, polyunsaturated fatty acids, ω-3, omega-3 fatty acids; ω-6, omega-6 fatty acids; TPC, total polyphenol content evaluated by Folin-Ciocalteu method.

**Table 3:** Correlation analysis between dietary markers and DNA damage in women and men.

	Women						Men					
	FPG-Sensitive Sites			H_2_O_2_-Induced Damage			FPG-Sensitive Sites			H_2_O_2_-Induced Damage		
Dietary Markers	Tau	Z	*p*-Level	Tau	Z	*p*-Level	Tau	Z	*p*-Level	Tau	Z	*p*-Level
Energy (kcal)	0.054	0.398	0.690	−0.060	−0.440	0.660	0.004	0.028	0.977	0.135	0.878	0.380
Total Carbohydrates (% of energy)	−0.052	−0.377	0.706	0.080	0.586	0.558	0.083	0.538	0.591	−0.135	−0.878	0.380
Simple Carbohydrates (% of energy)	0.040	0.293	0.769	−0.034	−0.251	0.802	−0.113	−0.735	0.462	−0.035	−0.226	0.821
Proteins (% of energy)	−0.123	−0.902	0.367	−0.014	−0.105	0.917	0.130	0.848	0.397	−0.052	−0.339	0.735
Animal Proteins (% of energy)	−0.057	−0.419	0.675	−0.040	−0.293	0.769	0.009	0.057	0.955	0.087	0.565	0.572
Vegetable Proteins (% of energy)	−0.046	−0.335	0.738	−0.006	−0.042	0.967	0.009	0.057	0.955	0.052	0.339	0.735
Total Lipids (% of energy)	0.100	0.734	0.463	−0.054	−0.398	0.690	−0.191	−1.243	0.214	0.234	1.526	0.127
SFA (% of energy)	0.109	0.796	0.426	0.046	0.335	0.738	−0.182	−1.187	0.235	0.139	0.904	0.366
MUFA (% of energy)	0.080	0.586	0.558	−0.006	−0.042	0.967	−0.304	−1.978	**0.048**	0.191	1.243	0.214
PUFA (%of energy)	0.052	0.377	0.706	0.000	0.000	1.000	−0.174	−1.130	0.258	0.148	0.961	0.337
ω-6 (%of energy)	0.052	0.379	0.705	−0.017	−0.126	0.899	−0.253	−1.646	0.100	0.148	0.965	0.335
ω-3 (%of energy)	−0.020	−0.149	0.881	0.020	0.149	0.881	−0.181	−1.177	0.239	0.181	1.177	0.239
Total Fibre (g/1000 kcal)	−0.023	−0.167	0.867	−0.269	−1.968	**0.049**	−0.161	−1.048	0.295	0.022	0.142	0.887
Cholesterol (mg)	0.029	0.212	0.832	0.353	2.584	**0.010**	−0.052	−0.341	0.733	0.200	1.306	0.192
Calcium (mg)	0.063	0.461	0.645	−0.137	−1.005	0.315	−0.087	−0.565	0.572	0.148	0.961	0.337
Iron (mg)	0.114	0.837	0.402	−0.097	−0.712	0.477	0.200	1.300	0.194	0.052	0.339	0.735
Vitamin B_12_ (mcg)	−0.036	−0.264	0.791	0.181	1.322	0.186	0.113	0.738	0.461	0.131	0.856	0.392
Vitamin C (mg)	−0.263	−1.926	0.054	0.057	0.419	0.675	−0.300	−1.954	0.051	0.170	1.105	0.269
Vitamin E (mg)	−0.172	−1.260	0.208	−0.046	−0.336	0.737	−0.312	−2.035	**0.042**	0.200	1.300	0.194
Vitamin B_1_ (mg)	−0.035	−0.253	0.801	−0.109	−0.800	0.424	−0.083	−0.540	0.589	0.031	0.199	0.842
Folates (mcg)	−0.391	−2.864	**0.004**	0.197	1.443	0.149	−0.229	−1.494	0.135	0.100	0.649	0.517
Vitamin B_6_ (mg)	−0.273	−1.998	**0.046**	0.043	0.315	0.752	−0.244	−1.589	0.112	0.288	1.873	0.061
Flavonoids (mg)	0.126	0.919	0.358	0.023	0.167	0.867	0.152	0.987	0.324	−0.004	−0.028	0.978
Lignans (mg)	0.009	0.064	0.949	0.155	1.138	0.255	−0.115	−0.751	0.453	0.177	1.156	0.248
Phenolic acids (mg)	−0.011	−0.084	0.933	0.114	0.835	0.404	−0.056	−0.367	0.714	−0.091	−0.592	0.554
Stilbenes (mg)	−0.060	−0.442	0.659	0.226	1.657	0.098	−0.233	−1.519	0.129	0.069	0.447	0.655
Other polyphenols (mg)	0.006	0.042	0.967	0.074	0.544	0.586	−0.299	−1.946	0.052	0.152	0.987	0.324
Total_Polyphenols (mg)	0.060	0.438	0.662	0.048	0.354	0.723	0.143	0.931	0.352	−0.100	−0.649	0.517
TPC_Folin (mg)	0.157	1.147	0.252	−0.003	−0.021	0.983	−0.195	−1.269	0.204	−0.126	−0.818	0.414

Legend: Regression and correlation analyses obtained by Spearman and Kendall test. Data reported in bold are statistically significant (*p* < 0.05); FPG, formamidopyrimidine DNA glycosylase; SFA, saturated fatty acids; MUFA, monounsaturated fatty acids; PUFA, polyunsaturated fatty acids, ω-3, omega-3 fatty acids; ω-6, omega-6 fatty acids; TPC, total polyphenol content.

**Table 4:** Correlation analysis between anthropometric, metabolic and clinical markers and DNA damage in the whole group of subjects.

Metabolic and Clinical Markers	FPG-Sensitive Sites			H_2_O_2_-Induced Damage		
	Tau	*Z*	*p*-Level	Tau	*Z*	*p*-Level
Age (y)	−0.169	−1.715	0.086	0.119	1.210	0.226
Weight (kg)	0.104	1.053	0.292	−0.210	−2.124	**0.034**
BMI (kg/m^2^)	0.063	0.638	0.524	−0.201	−2.034	**0.042**
SBP (mm Hg)	0.011	0.107	0.915	−0.163	−1.653	0.098
DBP (mm Hg)	0.142	1.435	0.151	−0.225	−2.283	**0.022**
Glucose (mg/dL)	0.039	0.399	0.690	−0.060	−0.608	0.543
Creatinine (mg/dL)	0.009	0.086	0.931	−0.044	−0.450	0.653
Uric Acid (mg/dL)	0.013	0.131	0.896	0.083	0.844	0.398
TC (mg/dL)	−0.130	−1.314	0.189	0.087	0.881	0.378
HDL-C (mg/dL)	−0.048	−0.487	0.626	−0.045	−0.452	0.651
TC/HDL (ratio)	−0.083	−0.845	0.398	0.133	1.345	0.179
LDL-C (mg/dL)	−0.123	−1.245	0.213	0.135	1.367	0.172
LDL/HDL (ratio)	−0.128	−1.302	0.193	0.166	1.682	0.093
TG (mg/dL)	−0.001	−0.009	0.993	0.037	0.372	0.710
AST (U/L)	0.029	0.292	0.770	−0.212	−2.151	**0.031**
ALT (U/L)	0.111	1.122	0.262	−0.252	−2.554	**0.011**
GGT (U/L)	0.113	1.149	0.251	−0.137	−1.392	0.164
Insulin (uU/mL)	−0.043	−0.432	0.665	−0.128	−1.297	0.194
HOMA Index	0.009	0.086	0.931	−0.119	−1.207	0.228
C-G index	0.119	1.207	0.228	−0.087	−0.879	0.379
Zonulin (ng/mL)	0.017	0.172	0.863	−0.063	−0.638	0.524
sICAM-1 (ng/mL)	0.027	0.276	0.783	0.022	0.224	0.823
sVCAM-1 (ng/mL)	−0.097	−0.983	0.326	0.133	1.345	0.179
CRP (mg/L)	0.085	0.862	0.389	0.172	1.741	0.082
TNF-α (pg/mL)	−0.107	−1.086	0.277	0.065	0.655	0.512
IL-6 (pg/mL)	−0.050	−0.509	0.611	0.076	0.767	0.443

Legend: Regression and correlation analyses obtained by Spearman and Kendall test. Data reported in bold are statistically significant (*p* < 0.05); BMI, body mass index; SBP, systolic blood pressure; DBP, diastolic blood pressure; TC, Total cholesterol, HDL-C, high density lipoprotein-cholesterol; LDL-C, low density lipoprotein-cholesterol; TG, triglycerides; AST, aspartate aminotransferase; ALT, alanine aminotransferase; GGT, gamma-glutamyl transpeptidase; HOMA index, homeostasis model assessment index; C-G index, Cockcroft–Gault index; sVCAM-1, vascular cells adhesion molecules-1; ICAM-1, intercellular adhesion molecules-1; CRP, C-reactive protein; TNF-α, tumour necrosis factor alpha; IL-6, interleukin-6.

**Table 5:** Correlation analysis between anthropometric, metabolic and clinical markers and DNA damage in women and men.

Markers	Women						Men					
	FPG-Sensitive Sites			H_2_O_2_-Induced Damage			FPG-Sensitive Sites			H_2_O_2_-Induced Damage		
	Tau	*Z*	*p*-Level	Tau	*Z*	*p*-Level	Tau	*Z*	*p*-Level	Tau	*Z*	*p*-Level
Age (y)	−0.095	−0.696	0.486	0.176	1.286	0.198	−0.205	−1.337	0.181	0.048	0.313	0.754
Weight (kg)	0.114	0.835	0.404	−0.171	−1.253	0.210	0.057	0.368	0.713	−0.143	−0.935	0.350
BMI (kg/m2)	0.048	0.354	0.723	−0.293	−2.147	**0.032**	0.108	0.705	0.481	−0.117	−0.761	0.446
SBP (mm Hg)	−0.178	−1.302	0.193	−0.136	−0.998	0.318	0.149	0.974	0.330	−0.202	−1.317	0.188
DBP (mm Hg)	0.081	0.590	0.555	−0.158	−1.159	0.246	0.156	1.018	0.309	−0.317	−2.066	**0.039**
Glucose (mg/dL)	0.069	0.504	0.614	0.040	0.294	0.769	0.013	0.085	0.932	−0.135	−0.882	0.378
Creatinine (mg/dL)	−0.080	−0.586	0.558	−0.029	−0.209	0.834	−0.066	−0.427	0.670	−0.118	−0.768	0.442
Uric Acid (mg/dL)	−0.020	−0.147	0.883	0.278	2.034	**0.042**	0.031	0.199	0.842	−0.100	−0.654	0.513
TC (mg/dL)	−0.120	−0.877	0.381	0.000	0.000	1.000	−0.121	−0.791	0.429	0.130	0.848	0.397
HDL-C (mg/dL)	0.000	0.000	1.000	−0.184	−1.348	0.178	−0.061	−0.399	0.690	0.079	0.513	0.608
TC/HDL (ratio)	−0.094	−0.688	0.491	0.236	1.730	0.084	−0.082	−0.536	0.592	0.108	0.705	0.481
LDL-C (mg/dL)	−0.160	−1.169	0.242	0.114	0.835	0.404	−0.061	−0.396	0.692	0.121	0.791	0.429
LDL/HDL (ratio)	−0.134	−0.980	0.327	0.288	2.106	**0.035**	−0.134	−0.874	0.382	0.126	0.818	0.414
TG (mg/dL)	0.003	0.021	0.983	0.060	0.440	0.660	−0.004	−0.028	0.977	0.004	0.028	0.977
AST (U/L)	0.070	0.514	0.607	−0.170	−1.243	0.214	−0.089	−0.581	0.562	−0.223	−1.451	0.147
ALT (U/L)	0.230	1.686	0.092	−0.190	−1.387	0.165	−0.058	−0.376	0.707	−0.325	−2.114	**0.035**
GGT (U/L)	0.124	0.909	0.363	0.043	0.317	0.751	0.035	0.227	0.820	−0.244	−1.589	0.112
Insulin (uU/mL)	0.009	0.063	0.950	−0.117	−0.855	0.393	−0.083	−0.538	0.591	−0.152	−0.991	0.322
HOMA Index	0.060	0.438	0.662	−0.054	−0.396	0.692	−0.013	−0.085	0.933	−0.169	−1.100	0.271
C-G index	0.094	0.688	0.491	−0.123	−0.896	0.370	0.091	0.592	0.554	−0.030	−0.197	0.844
Zonulin (ng/mL)	−0.020	−0.146	0.884	−0.088	−0.646	0.518	0.048	0.310	0.756	−0.022	−0.141	0.888
sICAM-1 (ng/mL)	0.048	0.354	0.723	0.151	1.105	0.269	−0.013	−0.085	0.933	−0.100	−0.649	0.517
sVCAM-1 (ng/mL)	−0.037	−0.271	0.786	0.077	0.563	0.574	−0.108	−0.705	0.481	0.255	1.664	0.096
CRP (mg/L)	0.014	0.104	0.917	0.174	1.272	0.203	0.160	1.043	0.297	0.229	1.494	0.135
TNF-α (pg/mL)	−0.014	−0.104	0.917	0.111	0.813	0.416	−0.212	−1.382	0.167	−0.022	−0.141	0.888
IL-6 (pg/mL)	−0.066	−0.479	0.632	0.105	0.771	0.441	0.056	0.367	0.714	0.039	0.254	0.800

Legend: Regression and correlation analyses obtained by Spearman and Kendall test. Data reported in bold are statistically significant (*p* < 0.05); BMI, body mass index; SBP, systolic blood pressure; DBP, diastolic blood pressure; TC, Total cholesterol, HDL-C, high density lipoprotein-cholesterol; LDL-C, low density lipoprotein-cholesterol; TG, triglycerides; AST, aspartate aminotransferase; ALT, alanine aminotransferase; GGT, gamma-glutamyl transpeptidase; HOMA index, homeostasis model assessment index; C-G index, Cockcroft–Gault index; sVCAM-1, vascular cells adhesion molecules-1; ICAM-1, intercellular adhesion molecules-1; CRP, C-reactive protein; TNF-α, tumour necrosis factor alpha; IL-6, interleukin-6; FPG, formamidopyrimidine DNA glycosylase.

## Data Availability

The data presented in this study are available on request from the corresponding author.

## Supplementary Materials

The following are available online at https://www.mdpi.com/article/10.3390/antiox10050730/s1, Figure S1: Levels of H_2_O_2_-induced DNA damage stratified by quartiles of body mass index (**A**), LDL/HDL ratio (**B**), triglycerides (**C**), HOMA-index (**D**) and C-reactive protein (**E**). Table S1: Baseline characteristics of subjects selected for the study, Table S2: Nutrient and polyphenol intake at baseline.
