# Supplementary material for: Association between Food Intake, Clinical and Metabolic Markers and DNA Damage in Older Subjects
Source: Antioxidants (Basel). 2021 May 6;10(5):730. doi: 10.3390/antiox10050730 (PMC8148130; doi:10.3390/antiox10050730)
Supplement: Supplementary file 1 [file antioxidants-10-00730-s001.zip › antioxidants-1189799-SI.pdf]

Figure S1. Levels of H2O2-induced DNA damage stratified by quartiles of body mass index (**A**), LDL/HDL ratio (**B**), triglycerides (**C**), HOMA-index (**D**) and C-reactive protein (**E**). Asterisks indicate statistically significant differences between quartiles ($p < 0.05$)

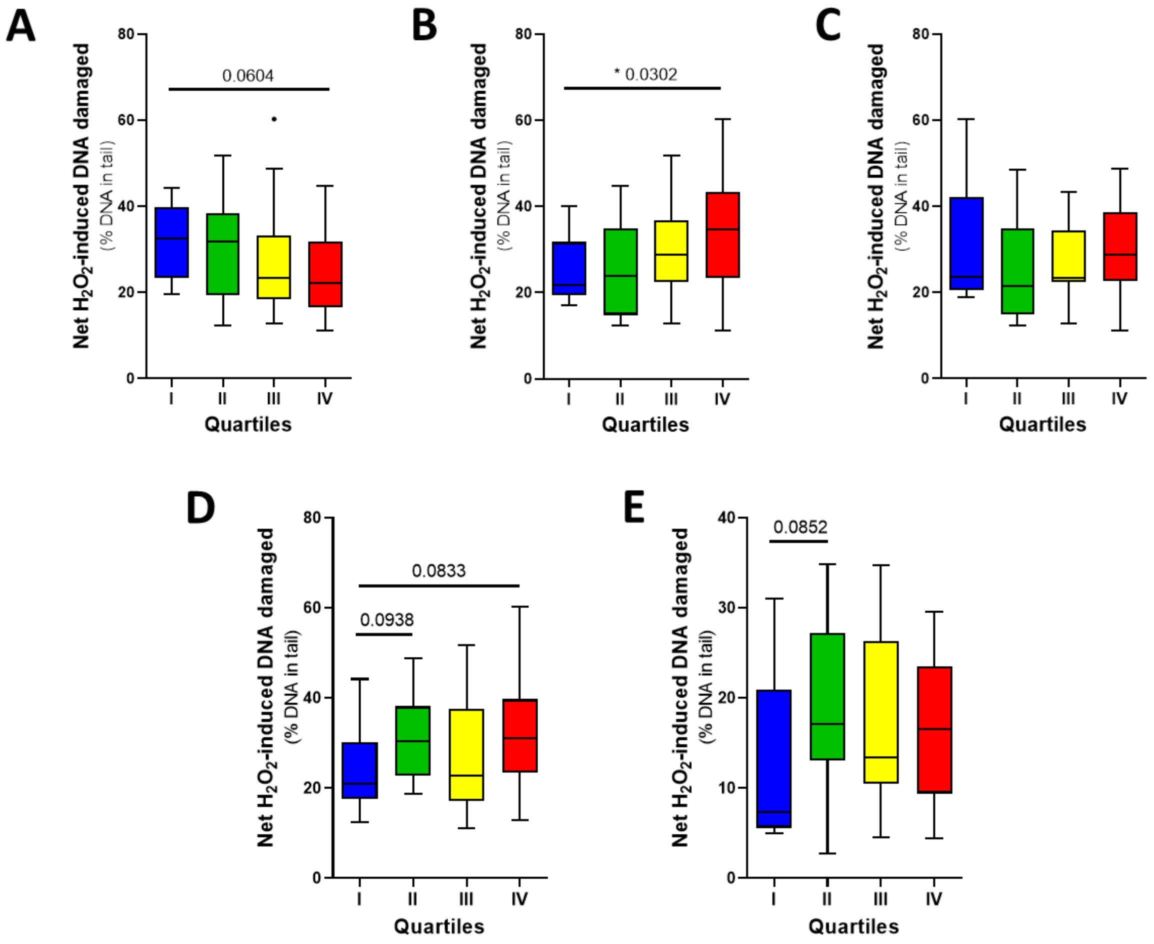

Table S1. Baseline characteristics of subjects selected for the study

Variables	Mean (SD)	Median (25°–75° Percentile)
Age (y)	78.0 ± 10.3	77 (71–86)
Sex (W/M)	27/22	–
Body weight (kg)	73.8 ± 13.8	74.0 (64.5–83.0)
BMI (kg/m²)	27.0 ± 5.5	26.4 (22.7–30.7)
SBP (mm Hg)	125.1 ±10.6	125 (120–130)
DBP (mm Hg)	76.1 ± 8.1	75 (70–80)
Glucose (mg/dL)	114.1 ± 68.5	95 (87–113)
Creatinine (mg/dL)	0.88 ± 0.30	0.87 (0.63–1.05)
Uric Acid (mg/dL)	5.6 ± 1.76	5.2 (4.3–6.6)
TC (mg/dL)	194.5 ± 48.6	194 (167–237)
HDL-C (mg/dL)	46.0 ± 14.7	45 (37–53)
LDL-C (mg/dL)	119.1 ± 35.7	120 (85–146)
TC/HDL-C (ratio)	4.5 ± 1.2	4.3 (3.5–5.4)
LDL/HDL-C (ratio)	2.7 ± 0.8	2.57 (2.08–3.45)
TG (mg/dL)	148 ± 95	117 (91–169)
AST (U/L)	17.9 ± 5.8	17 (14–22)
ALT (U/L)	13.5 ± 7.3	11 (8–19)
GGT (U/L)	38.7 ± 39.7	23 (18–46)
Insuline (uU/mL)	8.5 ± 6.5	6.2 (5.0–9.2)
HOMA index	2.9 ± 5.5	1.5 (1.2–2.5)
C-G index	75.4 ± 41.0	69.4 (54.7–82.3)
Zonulin (ng/mL)	42.5 ± 11.7	40.0 (35.5–49.2)
sVCAM-1 (ng/mL)	1238 ± 1714	968 (637–1293)
sICAM-1 (ng/mL)	56.3 ± 20.6	62.3 (44.2–65.4)
CRP (mg/L)	7.0 ± 8.1	3.5 (1.6–9.4)
TNF-α (pg/mL)	1.6 ± 1.2	1.2 (1.0–1.8)
IL-6 (pg/mL)	4.5 ± 4.2	3.1 (1.9–5.4)

Data are presented as mean ± standard deviation (SD) and median (25°-75° percentile); (n=49). W, women; M, men; BMI, body mass index; SBP, systolic blood pressure; DBP, diastolic blood pressure; TC, Total cholesterol, HDL-C, high density lipoprotein-cholesterol; LDL-C, low density lipoprotein-cholesterol; TG, triglycerides; AST, aspartate

aminotransferase; ALT, alanine aminotransferase; GGT, gamma-glutamyl transpeptidase; HOMA index, homeostasis model assessment index; C-G index, Cockcroft-Gault, sVCAM-1, vascular cells adhesion molecules-1; ICAM-1, intercellular cells adhesion molecules-1; CRP, C-reactive protein; TNF-α, tumour necrosis factor-alpha; IL-6, interleukin-6

Table S2. Nutrient and polyphenol intake at baseline

Variables	Mean ± SD	Median (IQR)
Energy (Kcal)	1584 ± 108	1587 (1528–1665)
Total carbohydrates (% of energy)	50.0 ± 2.7	50.8 (48.0–51.2)
Protein (% of energy)	17.8 ± 0.8	17.7 (17.3–18.4)
Animal proteins (% of energy)	12.0 ± 1.1	11.8 (11.3–12.8)
Vegetable proteins (% of energy)	5.7 ± 0.6	5.7 (5.4–5.8)
Total lipids (% of energy)	32.1 ± 2.3	31.5 (30.3–34.2)
SFA (% of energy)	8.7 ± 1.5	8.5 (7.9–9.0)
MUFA (% of energy)	16.3 ± 1.3	16.0 (15.4–16.9)
PUFA (% of energy)	3.2 ± 0.8	3.2 (3.0–3.3)
ω-3 (% of energy)	0.6 ± 0.2	0.6 (0.58–0.64)
ω-6 (% of energy)	2.5 ± 0.4	2.5 (2.3–2.6)
Total Fibre (g/1000 kcal)	11.2 ± 1.2	11.3 (10.6–12.1)
Cholesterol (mg)	207.8 ± 30.9	219 (187–228)
Calcium (mg)	804.6 ± 134.4	829.6 (708.8–886.4)
Iron (mg)	9.4 ± 0.9	9.3 (8.9-10.0)
Vitamin B_{12} (μg)	4.2 ± 1.0	4.3 (4.1–4.3)
Vitamin C (mg)	110.6 ± 56.9	98.8 (72.3–143.6)
Vitamin E (mg)	11.4 ± 2.9	11.8 (11.2–12.2)
Vitamin B_1 (mg)	0.8 ± 0.2	0.7 (0.7–0.9)
Folates (μg)	300.7 ± 74.8	310.9 (280–346)
Vitamin B_6 (mg)	1.5 ± 0.3	1.45 (1.35–1.58)
Flavonoids (mg)	181.5 ± 138.2	176.9 (68.2–216.1)
Lignans (mg)	0.79 ± 0.21	0.83 (0.64–0.85)
Other-Polyphenols (mg)	27.8 ± 4.3	29.1 (27.8–29.8)
Phenolic acids (mg)	131.0 ± 36.7	129.7 (116.5–145.4)
Stilbenes (mg)	0.04 ± 0.06	0.03 (0.03–0.03)

Total_Polyphenols (mg)	351.8 ± 159.5	338.4 (237.7–426.0)
TPC_Folin (mg)	661.1 ± 149.4	643.9 (555.7–732.0)

All data are expressed as mean ± standard deviation (SD), median and interquartile range (IQR); (n=49). SFA, saturated fatty acids; MUFA, monounsaturated fatty acids; PUFA, polyunsaturated fatty acids; ω-3, omega-3 fatty acids; ω-6, omega-6 fatty acids; TPC, total polyphenol content.
